# Supplementary material for: Establishment and Comparison of Detection Methods for Ricin and Abrin Based on Their Depurination Activities
Source: Toxins (Basel). 2025 Apr 3;17(4):177. doi: 10.3390/toxins17040177 (PMC12031163; doi:10.3390/toxins17040177)
Supplement: Supplementary file 1 [file toxins-17-00177-s001.zip › toxins-3554337-supplementary.pdf]

Supplementary Materials: Establishment and Comparison of Detection Methods for Ricin and Abrin Based on Their Depurination Activities

Lina Dong, Tingting Liu, Jiaxin Li, Cen Wang, Jing Lv, Jing Wang, Jinglin Wang, Shan Gao, Lin Kang and Wenwen Xin

Table S1. Results of affinity correlation constant determination of antibody.

	Conc(nM)	kon(1/Ms)	kdis(1/s)	KD (M)	RMax	Req	R2
B4	40	2.42E+05	2.03E-03	8.39E-09	2.527	2.0891	0.999
pAb-AT	40	8.19E+05	1.13E-05	1.38E-11	0.2075	0.2075	0.997

Note: Conc: toxin concentration; Kon: binding rate constant; Kdis: dissociation rate constant; KD: affinity constant (dissociation equilibrium constant); RMax: the maximum theoretical value of the nanobody binding signal; Req: the signal value when the binding signal of the nano antibody reaches equilibrium; R^2: Correlation coefficient of the simulated curve.

Table S2. Single stranded DNA substrate

Substrate name	Nucleotide sequence (5'—3')	Notes
DNA14-2GA	CGCGCGAGAGCGCG	
DNA20T-2GA	TATATATAGAGATATATATA	The neck ring structure contains two GA structures
DNA24T-2GA	TATATATATAGAGATATATATATA	
DNA28T-2GA	TATATATATATAGAGATATATATATATA	
DNA28T-4GA	TATATATATAGAGAGAGATATATATATA	Neck ring structure, GA quantity increases
DNA30T-5GA	TATATATATAGAGAGAGAGATATATATATA	
DNA32T-6GA	TATATATATAGAGAGAGAGAGATATATATATA	
DNA34T-7GA	TATATATATAGAGAGAGAGAGAGATATATATATA	
DNA10A	AAAAAAAAAA	It contains only adenine, and the amount of adenine increases
DNA15A	AAAAAAAAAAAAAAA	
DNA20A	AAAAAAAAAAAAAAAAAAAA	
DNA25A	AAAAAAAAAAAAAAAAAAAAAAAAA	
DNA30A	AAAAAAAAAAAAAAAAAAAAAAAAAAAAAA	

Table S3. Single stranded RNA substrates

Substrate name	Nucleotide sequence (5'—3')	Notes
RNA14-2GA	CGCGCGAGAGCGCG	
RNA20U-2GA	UAUAUAUAGAGAUAUAUAUA	The neck ring structure contains two GA structures
RNA24U-2GA	UAUAUAUAUAGAGAUAUAUAUAUA	
RNA28U-2GA	UAUAUAUAUAUAGAGAUAUAUAUAUAUA	
RNA28U-4GA	UAUAUAUAUAGAGAGAGAUAUAUAUAUA	
RNA30U-5GA	UAUAUAUAUAGAGAGAGAGAUAUAUAUAUA	Neck ring structure, GA quantity increases
RNA32U-6GA	UAUAUAUAUAGAGAGAGAGAGAUAUAUAUAUA	
RNA34U-7GA	UAUAUAUAUAGAGAGAGAGAGAGAUAUAUAUAUA	
RNA10A	AAAAAAAAAA	
RNA15A	AAAAAAAAAAAAAAA	
RNA20A	AAAAAAAAAAAAAAAAAAAA	It contains only adenine, and the amount of adenine increases
RNA25A	AAAAAAAAAAAAAAAAAAAAAAAAA	
RNA30A	AAAAAAAAAAAAAAAAAAAAAAAAAAAAAA	

Table S4. Double-stranded DNA substrates

Substrate name	Nucleotide sequence (5'—3')	Notes
dDNA25	GCGCGCGACAGACAGACAGCGCGG<br>C	
dDNA25-2T	GCGCGCGCGCGATATCGCGCGGCG<br>C	The length of the chain remained unchanged, and the thymine content increased
dDNA25-4T	GCGCGCGCGATATATATCGCGCGC<br>G	
dDNA25-6T	GCGCGCGATATATATATATGCGCGC	
dDNA43AT	TATATATATATATATATATATATAT<br>ATATATATATATATATATATATATA<br>TATATATATATATATATATATATAT<br>ATATATATATA	
dDNA45AT	TATATATATATATATATATATATAT<br>ATATATATATATATATATATATATA<br>TATATATATATATATATATATATAT<br>ATATATATATATATA	It only contains adenine and thymine, and the content of adenine and thymine increases
dDNA47AT	TATATATATATATATATATATATAT<br>ATATATATATATATATATATATATA<br>TATATATATATATATATATATATAT<br>ATATATATATATATATATA	

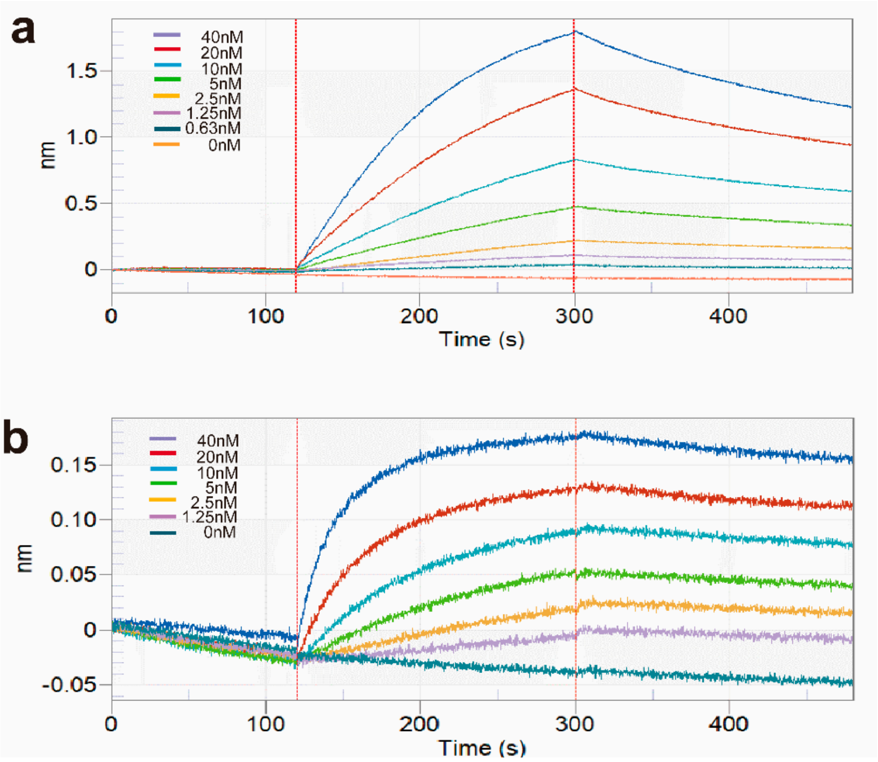

Figure S1. Flow chart of interaction binding dissociation of nanobody

(a) The binding dissociation diagram of RT and nanobody B4; (b) The binding dissociation diagram of AT and AT polyclonal antibody.

A

Start codes

MGSDKIIHLTDDSFDTDVLKADGAILVDFWAEWCGPCKMIAPILDEIADEYQGKLTVAKLNIDQNPGTAPKYGIRGIP

Trx-tag

TLLLFKNGEVAATKVGALSKGQLKEFLDANLAGGGGSGGGGSGGGGSEVQLQASGGGLVQAGGSLRLSCVHSGSPLRS

Linker

SAMAWFRQAPGKDREFVATINFSGSLAKYTDSVKGRFTISRDNDQNTVYLQMNSLKAEDAAVYYCAAAPAWDRLEYAP

B4

RAASDFVSWGPGTQVTVFAEPKTPKPQPHHHHHH

His-tag

B

Start codes

MGSDKIIHLTDDSFDTDVLKADGAILVDFWAEWCGPCKMIAPILDEIADEYQGKLTVAKLNIDQNPGTAPKYGIRGIP

Trx-tag

TLLLFKNGEVAATKVGALSKGQLKEFLDANLAGGGGSGGGGSGGGGSDYQLQASGGGLAQAGGSLRLSCAYSGDTVND

Linker

YAMAHFRQAPGKGREFVAAIRARGGGTEYLDSVKGRFTISRDNGENTAYLQMDNLQPDDTALYFCALAMGGYAYRAFE

H1

RYSVRGQGTQVTVSSEPKTPKPQPAAAGSGSGGGSSGGGSGASGSEVQLQASGGGLVQAGGSLRLSCVHSGSPLRSSA

Linker

MAWFRQAPGKDREFVATINFSGSLAKYTDSVKGRFTISRDNDQNTVYLQMNSLKAEDAAVYYCAAAPAWDRLEYAPRA

B4

ASDFVSWGPGTQVTVFAEPKTPKPQPGGGSHHHHHH

His-tag

C

Start codes

MGSDKIIHLTDDSFDTDVLKADGAILVDFWAEWCGPCKMIAPILDEIADEYQGKLTVAKLNIDQNPGTAPKYGIRGIP

Trx-tag

TLLLFKNGEVAATKVGALSKGQLKEFLDANLAGGGGSGGGGSGGGGSSGGGLVQAGGSLRLSCAASGSAVSDSFSTYA

Linker

ISWHRQAPGKQREWIAGISNRGATSYRDSVKGRFTISRDNAKNTVYLQMNNLKPEDTGVYYCEPWPREGLGGGQGTQV

RTB-G5

TVSSGGGGSGGGGSGGGGSSGGAVVQPGGSLRLSCATSGFTFSDDRMSWARQAPGKGLEWVSGISTASEGFATLYAPS

Linker RTB-B7

VKGRFTISRDNAKHMLYLQMDTLKPEDTAVYYCLRGVFFRTNIPPEVLRGQGTQVTVSSHHHHHH

His-tag

D

Start codes

MGSDKIIHLTDDSFDTDVLKADGAILVDFWAEWCGPCKMIAPILDEIADEYQGKLTVAKLNIDQNPGTAPKYGIRGIP

Trx-tag

TLLLFKNGEVAATKVGALSKGQLKEFLDANLAGGGGSGGGGSGGGGSSGGGLVQAGGSLRLSCAASGSAVSDSFSTYA

Linker

ISWHRQAPGKQREWIAGISNRGATSYRDSVKGRFTISRDNAKNTVYLQMNNLKPEDTGVYYCEPWPREGLGGGQGTQV

RTB-G5

TVSSGGGGSGGGGSGGGGSTGGDLVQPGGSLRLSCAASGSSFSRAAVGWYRQAPGKEREWVARLASGDMTDYTESVRG

Linker RTB-B9

RFTISRDNAKHTVYLQMDNLKPEDTAVYYCKARIPPYYSIEYWGKGTRVTVSSHHHHHH

His-tag

E Start codes

MGSDKIIHLTDDSFDTDVLKADGAILVDFWAEWCGPCKMIAPILDEIADEYQGKLTVAKLNIDQNPGTAPKYGIRGIP

Trx-tag

TLLLFKNGEVAATKVGALSKGQLKEFLDANLAGGGGSGGGGSGGGGSEVQLQASGGGLVQAGGSLRLSCVVSGLSVGL

Linker

NPVAWYRQAPGQQRDWVARMSMRGLTDYPDSLKGRAIISRDNAKNTVSLQMDSLKPEDTAVYFCKVGLSYWGQGTQVT

Abr5

VSSHHHHHH

His-tag

F Start codes

MGSDKIIHLTDDSFDTDVLKADGAILVDFWAEWCGPCKMIAPILDEIADEYQGKLTVAKLNIDQNPGTAPKYGIRGIP

Trx-tag

TLLLFKNGEVAATKVGALSKGQLKEFLDANLAGGGGSGGGGSGGGGSEVQLVESGGGLVQAGRSLILSCSASGGIFTN

Linker

MGMGWYRQAPGKQREVVAQITNRGRTNYGESVKGRFTISRDNAQRMAYLQMNSLKPEDTAVYYCRTNYGGRGYWGQGT

Abr10C

QVTVSSHHHHHH

His-tag

G Start codes

MGSDKIIHLTDDSFDTDVLKADGAILVDFWAEWCGPCKMIAPILDEIADEYQGKLTVAKLNIDQNPGTAPKYGIRGIP

Trx-tag

TLLLFKNGEVAATKVGALSKGQLKEFLDANLAGGGGSGGGGSGGGGSDVQLQASGGGLVRAGGSLRLSCAVSGLTVSH

Linker

APSAWYRQAPGKQRDWVARLSMRGLTDYEDSVKGRFTISRDNAKNTVYLQMDSLKPEDTAVYFCKAGLNYWGQGIQVT

Abr11

VSSHHHHHH

His-tag

H Start codes

MGSDKIIHLTDDSFDTDVLKADGAILVDFWAEWCGPCKMIAPILDEIADEYQGKLTVAKLNIDQNPGTAPKYGIRGIP

Trx-tag

TLLLFKNGEVAATKVGALSKGQLKEFLDANLAGGGGSGGGGSGGGGSEVQLVESGGGLVQAGRSLILSCSASGGIFAN

Linker

MGMGWYRQAPGKQREVVAQITNRGRTNYGESVKGRFTISRDNAQRMAYLQMNSLKPEDTAVYYCRTNYGGRDYWGQGT

Abr3E

QVTVSSHHHHHH

His-tag

Figure S2. Design of ricin and abrin nanobody sequences.

(a) Sequence design of B4 nanobody; (b) Sequence design of 21-B4 nanobody; (c) Sequence design of G5/B7 nanobody; (d) Sequence design of G5/B9 nanobody; (e) Sequence design of Abr5 nanobody; (f) Sequence design of Abr10C nanobody; (g) Sequence design of Abr11 nanobody; (h) Sequence design of Abr3E nanobody.
